# Delayed Spontaneous Pneumocephalus After Ventriculoperitoneal Shunt Surgery: A Successful Conservative Treatment

**DOI:** 10.7759/cureus.102938

**Published:** 2026-02-04

**Authors:** Eliza Maria Bertolaccini Scolin, José Orlando de Melo Junior, Paulo José Da Mata Pereira, Paulo Niemeyer Filho

**Affiliations:** 1 Neurosurgery Department, Paulo Niemeyer State Brain Institute, Rio de Janeiro, BRA

**Keywords:** negative intracranial pressure, pneumocephalus, siphon effect, ventriculoperitoneal shunt, vp shunt complication

## Abstract

Pneumocephalus is defined as the presence of intracranial air and is a rare but potentially significant complication following ventriculoperitoneal (VP) shunt placement. Delayed presentations are uncommon and remain poorly described in the literature. The report demonstrates the case of a 74-year-old male patient who developed delayed spontaneous intraventricular pneumocephalus 10 months after VP shunt insertion in September 2022 using a high-pressure fixed valve for obstructive hydrocephalus secondary to a tectal plate lesion. The patient presented in July 2023 with posture-dependent frontal headache, tinnitus, and bruit hydroaérique, without fever, infection, or neurological deterioration. Computed tomography revealed intraventricular air and a small air focus adjacent to the mastoid tegmen, suggestive of an occult skull base defect. The presumed mechanism involved sustained negative intracranial pressure related to the siphon effect of the shunt system, facilitating air entry through a pre-existing osteodural defect. Given the patient’s clinical stability and absence of infection or shunt malfunction, a conservative management strategy was adopted. Complete clinical and radiological resolution was observed within two weeks, with no recurrence during 12 months of follow-up. This case highlights a rare delayed complication of VP shunting and demonstrates that conservative management may be a safe and effective option in carefully selected, clinically stable patients, after exclusion of infection and shunt dysfunction. It contributes to the limited existing literature on non-invasive treatment strategies for delayed pneumocephalus.

## Introduction

Pneumocephalus, defined as the presence of intracranial air, is an uncommon but potentially serious complication following ventriculoperitoneal (VP) shunt placement [1]. Although most cases arise in the context of trauma, neurosurgical procedures, or infection by gas-producing organisms, VP shunt-associated pneumocephalus represents a distinct pathophysiological entity driven by alterations in intracranial pressure dynamics [2,3]. The siphon effect generated by the shunt system can create negative intracranial pressure, predisposing to air entry through pre-existing or acquired osteodural defects that communicate with air-containing cavities such as the paranasal sinuses, middle ear, or mastoid cells. Additional factors-including positive pressure ventilation, Valsalva maneuvers, and elevated intra-abdominal pressure-may exacerbate this pressure gradient and facilitate retrograde air migration through the shunt system or cranial defects [4,5]. While acute pneumocephalus is well recognized in the immediate postoperative or post-traumatic setting, delayed presentations are exceedingly rare and pose a diagnostic and therapeutic challenge.

Computed tomography (CT) remains the diagnostic gold standard, offering high sensitivity for intracranial air detection and allowing evaluation of ventricular morphology and potential sources of air ingress [6]. Management strategies range from conservative measures-such as bed rest, supplemental oxygen, and adjustment of shunt valve pressure-to surgical intervention involving decompression and dural defect repair in cases of tension pneumocephalus or neurological deterioration [7-14].

This case describes a rare instance of delayed spontaneous pneumocephalus occurring 10 months after VP shunt placement, likely resulting from a shunt-induced siphon effect in the presence of an occult skull base defect. It underscores the importance of recognizing this uncommon complication and highlights the effectiveness of conservative management in a clinically stable patient without signs of infection or cerebrospinal fluid (CSF) leakage.

## Case presentation

**Figure 1:**
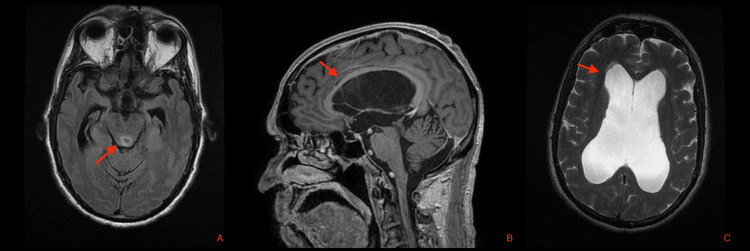
Preoperative brain MRI findings. (A) Axial FLAIR sequence showing an expansive lesion in the tectal plate (red arrow) compressing the cerebral aqueduct. (B) Sagittal T1-weighted contrast-enhanced image showing supratentorial ventricular enlargement consistent with obstructive hydrocephalus (red arrow). (C) Axial T2-weighted image demonstrating supratentorial hydrocephalus (red arrow). MRI: magnetic resonance imaging; FLAIR: fluid-attenuated inversion recovery

A 74-year-old male patient presented in September 2022 with a one-year history of progressive cognitive and gait disturbances. According to the patient and his family members, he reported “difficulty remembering places and getting oriented,” “trouble walking with frequent imbalance,” and “increasing urinary urgency with occasional incontinence.” These symptoms had progressively worsened over the preceding six months, resulting in significant functional decline and loss of independence in activities of daily living. There was no history of headache, fever, seizures, head trauma, or visual disturbances at the time of presentation. On neurological examination, the patient was alert but demonstrated temporospatial disorientation, a broad-based gait with marked gait apraxia, and impaired postural stability. Cranial nerve examination was unremarkable, and no focal motor or sensory deficits were identified. Brain magnetic resonance imaging (MRI) revealed an expansive lesion compressing the cerebral aqueduct, suggestive of a tectal plate glioma, associated with obstructive hydrocephalus. The patient subsequently underwent VP shunt placement in September 2022 using a high-pressure fixed valve. The immediate postoperative course was uneventful, and a head CT scan obtained on postoperative day one showed no abnormalities. He was discharged on postoperative day two with complete resolution of symptoms. The tectal lesion was managed conservatively with serial MRI follow-up (Figure 1).

**Figure 2:**
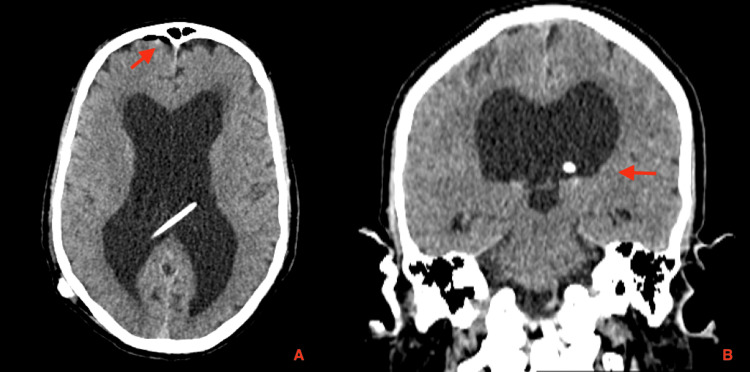
Postoperative head CT scan demonstrating appropriate placement of the ventricular catheter within the right lateral ventricle. (A) Axial CT image showing the ventricular catheter tip in optimal position, with a small amount of right frontal subdural pneumocephalus likely related to the ventricular puncture (red arrow). (B) Coronal CT image confirming correct intraventricular positioning. CT: computed tomography

**Figure 3:**
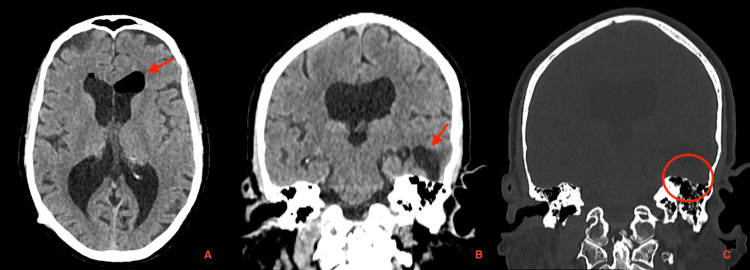
Head CT scan demonstrating spontaneous intraventricular pneumocephalus (red arrow, image A) and a region of left temporal encephalomalacia with a cystic, CSF-filled appearance (red arrow, image B). A small air bubble is observed adjacent to the mastoid tegmen, suggesting a possible mastoid bone defect (red circle, image C). CT: computed tomography; CSF: cerebrospinal fluid

Ten months postoperatively, the patient returned with a two-week history of daily frontal headaches (rated 6/10), which were posture-dependent and worsened in the upright position, associated with progressively worsening tinnitus and a bruit hydroaérique, without identifiable triggers. Neurological examination was non-focal, with no signs of infection. The VP shunt pump was compressible and functioning appropriately; laboratory studies were normal, and the patient was afebrile, with no evidence of infection or shunt malfunction. A head CT scan revealed spontaneous intraventricular pneumocephalus (Figure 2). Additionally, a previously undocumented area of left temporal encephalomalacia with a cystic CSF-filled appearance was identified. A small air bubble was noted adjacent to the mastoid tegmen, suggestive of a possible mastoid bone defect (Figure 3).

**Figure 4:**
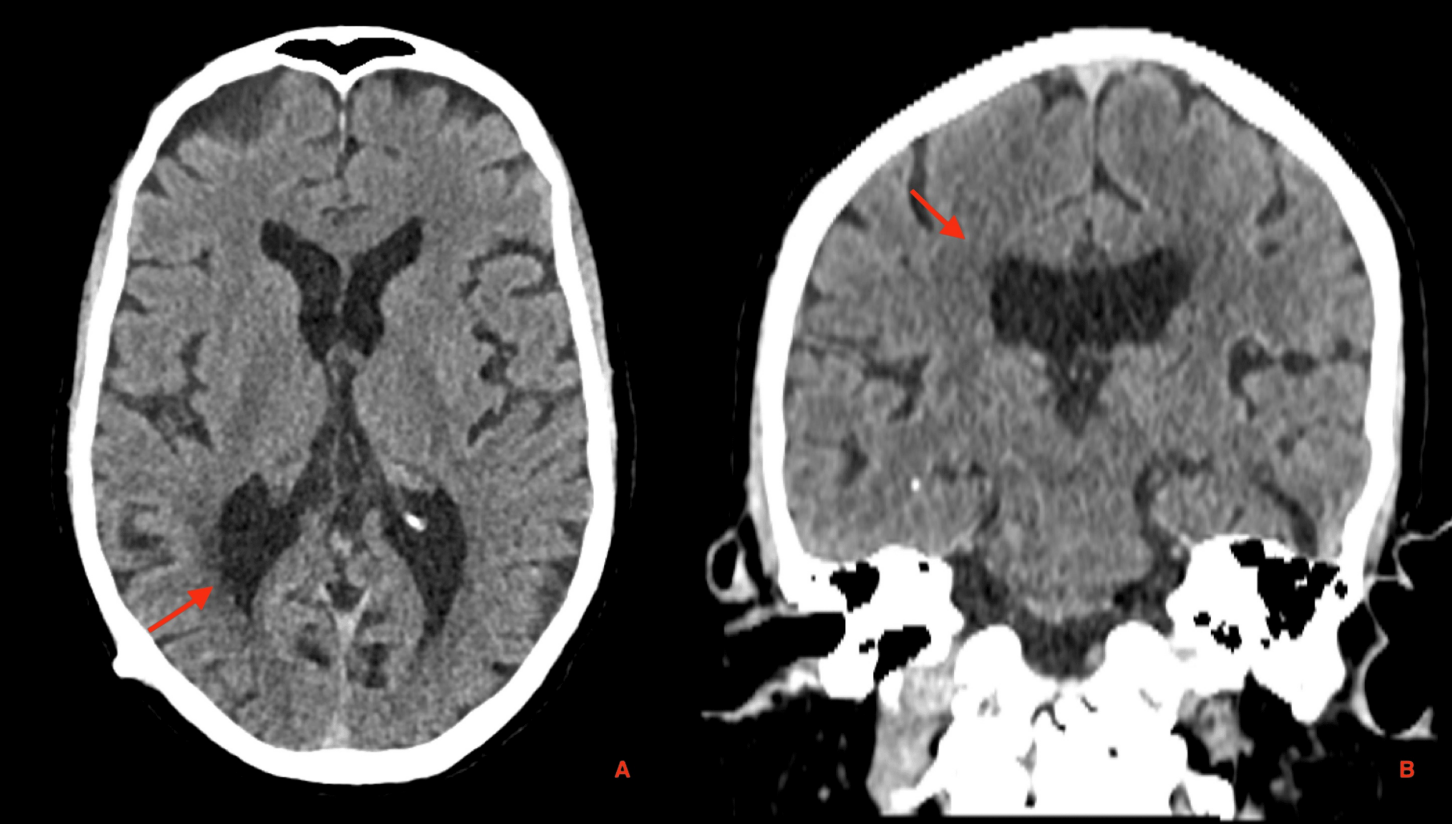
Two-week follow-up cranial CT demonstrating complete resolution of the previously observed intraventricular pneumocephalus and left temporal encephalomalacia. (A) Axial and (B) coronal reconstructions show normal ventricular configuration and absence of intracranial air, confirming full radiological resolution after conservative management (red arrow). CT: computed tomography

**Figure 5:**
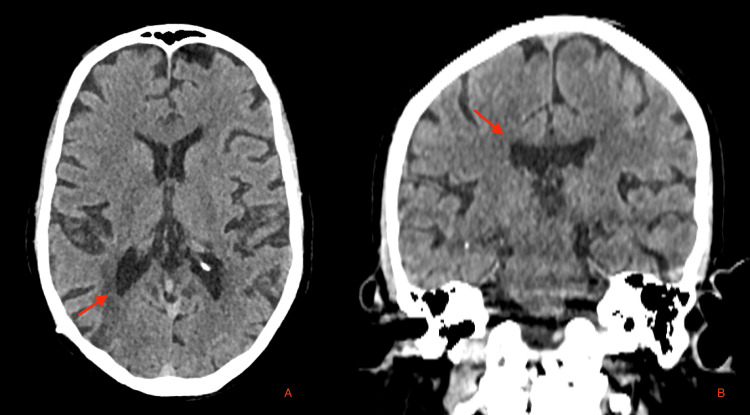
One-year follow-up cranial CT images showing maintained complete resolution of the previously observed intraventricular pneumocephalus and left temporal encephalomalacia. (A) Axial and (B) coronal reconstructions demonstrate normal ventricular morphology and no evidence of intracranial air (red arrow). CT: computed tomography

The patient denied recent head trauma, though a past history of head trauma could not be safely excluded due to cognitive impairment and social vulnerability. Due to the absence of infection and neurologic decline, a conservative approach was adopted. The patient was monitored with weekly clinical and radiological assessments. Within two weeks, follow-up CT imaging demonstrated complete resolution of the pneumocephalus (Figures 4, 5). The patient has improved his symptoms and has shown no recurrence during one year of follow-up.

## Discussion

Pneumocephalus refers to the presence of air within the cranial cavity and may occur in various compartments, including the brain parenchyma, ventricular system, epidural, subdural, or subarachnoid spaces [1,2]. It is classified as acute when occurring within 72 hours of onset and delayed or chronic when detected after this period [1,3]. Historically, pneumocephalus was first described by Lecat in 1741 in a patient with a skull fracture. Luckett, in 1913, was the first to identify ventricular pneumocephalus on skull radiography, and Wolff introduced the term "pneumoencephalus" in 1914 [2,4]. During World War I (1914-1918), multiple cases were documented, often secondary to penetrating cranial injuries [2].

Pneumocephalus is a multifactorial condition with a wide range of etiologies, resulting from traumatic brain injury, surgical procedures including VP shunting, congenital skull base defects, infections by gas-producing organisms (*Klebsiella* spp., *Bacteroides* spp., *Escherichia coli*, *Peptostreptococcus* spp., *Fusobacterium* spp., and *Streptococcus pyogenes*), or even spontaneous occurrence [2-4].

Symptoms depend on both the volume and the location of the intracranial air [4]. Most cases are benign, asymptomatic, and self-limiting [1,4]. However, red flags include scalp or tympanic membrane lacerations, CSF fistulas, persistent postoperative headache, seizures, meningitis, tinnitus, and the rarely reported bruit hydroaérique-a splashing sound perceived during head movement, considered pathognomonic of pneumocephalus [4,5]. Non-contrast cranial CT is the gold standard for diagnosis, with the capacity to detect air volumes as small as 0.55 mL [4,6].

Most cases of pneumocephalus resolve with conservative measures, including bed rest, head elevation, avoidance of Valsalva maneuvers, and administration of supplemental oxygen [1,4,7]. Management of pneumocephalus in patients with VP shunts depends on the underlying etiology, which may include infection, shunt dysfunction, trauma, or skull base defects [4,7,8]. Standard approaches typically involve shunt revision, temporary adjustment of valve pressure, CSF diversion, repair of osseous defects, or antibiotic therapy when infection is present [2,8-14]. However, there is limited documentation in the literature regarding non-invasive management strategies for delayed pneumocephalus in clinically stable patients post-VP shunt [5,9,14].

In the present case, delayed pneumocephalus developed 10 months after VP shunt placement, in the absence of confirmed trauma or infection. This delayed presentation, together with CT findings suggestive of a possible mastoid bone defect, supports a pathophysiological mechanism related to sustained negative intracranial pressure, most likely attributable to the siphon effect of the shunt in combination with a previously unrecognized skull base defect [5,9,11]. Radiological evidence of a small air bubble in the mastoid region, along with the presence of a high-pressure fixed valve, further supports this hypothesis [5,11]. Sustained negative intracranial pressure may persist over time, predisposing the intracranial compartment to air entry through pre-existing osteodural defects [9,11,13]. In this setting, negative pressure not only facilitates air ingress but may also impair its clearance, allowing progressive accumulation of intracranial air even in the absence of trauma or infection [9,14]. Although no traumatic event was reported, a minor head injury could not be definitively excluded given the patient’s cognitive impairment and social vulnerability.

Given the patient’s clinical stability, absence of infection, and spontaneous onset of symptoms, a conservative management approach was adopted. This strategy included no adjustment of the shunt valve pressure, no surgical correction of the suspected skull base defect, and no administration of prophylactic antibiotic therapy. Similar conservative strategies have been reported in selected cases of delayed pneumocephalus without neurological deterioration or evidence of infection [5,9]. The patient was managed with close outpatient follow-up, education regarding warning signs, and recommendations to avoid activities that could increase intracranial pressure. Complete clinical and radiological resolution was observed within two weeks, with sustained symptom remission and no recurrence during 12 months of follow-up.

This case supports the consideration of individualized conservative management strategies for delayed pneumocephalus in selected patients with CSF shunting, as an alternative to more commonly reported approaches such as external drainage for infectious etiologies in conjunction with antibiotic therapy [10], temporary valve adjustment [11,12], or surgical repair of bone defects and CSF fistulas [9,13,14], highlighting the importance of individualized management strategies.

## Conclusions

Delayed pneumocephalus following VP shunt placement is a rare but recognized complication. In the present case, air entry into the cranial cavity was likely facilitated by a combination of negative intracranial pressure related to the shunt siphon effect and a pre-existing skull base defect. Conservative management, without valve revision, proved effective in a clinically stable patient with no signs of CSF leakage or infection, under close outpatient follow-up. This case highlights the potential role of conservative treatment in carefully selected patients, after exclusion of infection, shunt malfunction, or intracranial hypertension, and underscores the importance of individualized management strategies for delayed pneumocephalus associated with CSF shunting.
